# Spontaneous Bacterial Peritonitis Secondary to Salmonella spp. in a Patient With Decompensated Liver Cirrhosis

**DOI:** 10.7759/cureus.15432

**Published:** 2021-06-03

**Authors:** Katia El Jurdi, Ali Taleb, William J Salyers

**Affiliations:** 1 Internal Medicine, University of Kansas School of Medicine, Wichita, USA

**Keywords:** decompensated cirrhosis, spontaneous bacterial peritonitis, ascitic fluid culture, salmonella enterica, ascitic fluid analysis

## Abstract

Spontaneous bacterial peritonitis (SBP) is a common and serious complication of cirrhosis, with gram-negative bacteria being the culprit in most cases. SBP secondary to *Salmonella spp*. is rare. Here, we report a case of *Salmonella* *enterica* SBP in a patient with decompensated cirrhosis, diagnosed via paracentesis coupled with ascitic fluid analysis and culture. A high index of suspicion must be maintained for atypical causes of SBP, with prompt initiation of treatment.

## Introduction

Spontaneous bacterial peritonitis (SBP) is a common and serious complication of cirrhosis, which occurs in 10%-30% of patients with cirrhosis [1]. It is defined as an ascitic fluid infection in the absence of an intra-abdominal surgically treatable source of infection and should be suspected in patients with cirrhosis who develop signs and symptoms such as fever, abdominal pain and altered mental status. SBP is diagnosed by the presence of neutrophilic leukocytosis (greater than 250/mm^3^) in the peritoneal fluid [1]. Enteric bacterial organisms that translocate from the gut are implicated in the pathological process. Common organisms include *Escherichia coli*, *Klebsiella pneumoniae* and *Streptococcus* species. *Salmonella spp*. is a rare and atypical cause, with a few cases reported worldwide [2-8]. Here, we report a case of SBP due to *Salmonella enterica* in a patient with decompensated cirrhosis.

## Case presentation

A 63-year-old man presented with abdominal distension, worsening jaundice and lower extremity swelling over the previous 48 hours. Past medical history included Hepatitis C cirrhosis, upper gastrointestinal (GI) bleed secondary to esophageal varices and hepatocellular carcinoma (HCC). HCC, with metastasis to the hilar lymph nodes and lungs, was found to be unresectable and the patient was not a candidate for a liver transplant. He had been treated with lenvatinib as first-line, then transitioned to sorafenib and trans-catheter arterial chemo-embolization (TACE). Home medications included furosemide and spironolactone. He had no previous history of SBP and was not on prophylactic antibiotics. Social history was positive for previous tobacco use 15 years prior. There was no history of chronic liver disease or GI malignancies in the family.

**Figure 1:**
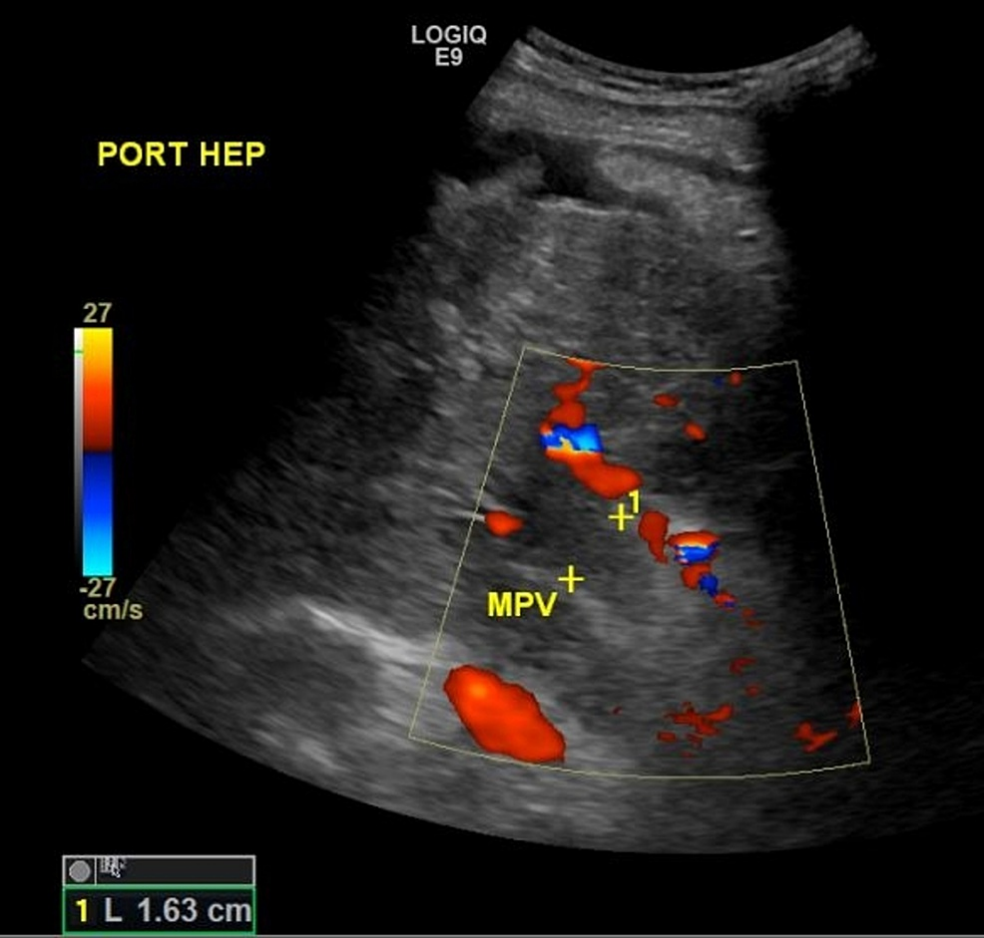
Ultrasound of the abdomen with the absence of color flow or spectral Doppler signal within the portal vein. The portal vein was occluded and measured 1.63 cm. PORT HEP - hepato-portal circulation MPV - Main portal vein

On exam, vital signs were within normal limits. The patient's weight was 78 kg and BMI was 27 kg/m^2^. Physical exam revealed scleral icterus, tense ascites and bilateral pitting edema in the lower extremities. No asterixis was noted. Exam of other organ systems was nonrevealing. Laboratory testing showed an elevated white blood cell count of 18.8 x 10^9^/L, low hemoglobin of 11.3 g/dL, a platelet count of 200 x 10^9^/L, elevated International normalized ratio of 1.9, low sodium of 123 mmol/L, elevated potassium of 5.5 mmol/L, low albumin of 1.6 g/dL, and elevated C- reactive protein level of 70 mg/L. Liver function enzymes were elevated: aspartate transaminase 129 U/L, alanine aminotransferase 71 U/L, and bilirubin 6.3 mg/dL. Ammonia level was elevated at 58 mg/L. Model for end-stage liver disease sodium (MELD-Na) score was 29, corresponding to 90-day mortality of 20%. Ultrasound of the abdomen demonstrated features of a cirrhotic liver and a large volume of ascites. The portal vein was occluded and measured 1.63 cm. There was cavernous transformation seen with color flow Doppler in the region of the portal vein (Figure 1). The patient underwent an ultrasound-guided, large-volume paracentesis and a total of 5.0 L of clear straw-colored fluid was removed. Peritoneal fluid cytology was negative for malignant cells. Ascitic fluid analysis revealed albumin less than 0.6 g/dL, total protein of 1.6 g/dL, lactate dehydrogenase of 182 U/L, and glucose of 43 mg/dL. Ascitic fluid polymorphonuclear neutrophil (PMN) count was 2,983, prompting empiric IV ceftriaxone therapy for SBP. Serum ascitic albumin gradient was >1.1 g/dL, consistent with ascites secondary to portal hypertension in the setting of cirrhosis and portal vein thrombosis.

Peritoneal fluid cultures were obtained. The culture was grown on a MacConkey agar plate and then confirmed with an API-20E test (BioMerieux, Inc., France). The test was positive for *S. enterica*, susceptible to ceftriaxone, levofloxacin and trimethoprim/sulfamethoxazole. Therefore, IV ceftriaxone was continued, with a good clinical response. The patient received IV albumin 25% infusion on day 1 and day 3 post paracentesis. The patient needed repeat paracentesis on day 3 and day 6, with the removal of another 5.0 L and 3.1 L of fluid, respectively. Repeat peritoneal cultures were negative, confirming response to antibiotic treatment. The patient had an uneventful course during hospitalization and completely recovered from the SBP, with the resolution of abdominal pain. He was transitioned to oral SBP prophylaxis therapy (trimethoprim/sulfamethoxazole daily) upon discharge.

## Discussion

*Salmonella spp.* is a pathogenic gram-negative bacteria predominately found in the intestinal lumen. Infection is not common in the United States and is usually manifested as limited gastroenteritis only [9]. Rarely does it cause SBP in individuals with decompensated cirrhosis. A review of the English literature revealed only a few reported cases of *Salmonella spp.* causing SBP in cirrhotic patients, most of which were reported outside the US [2-8]. This case presented was diagnosed with ascitic fluid studies and culture and subsequently treated with standard therapy based on current guidelines for SBP [10].

The opsonic (anti-microbial) activity in an ascitic fluid depends on the protein content. Low-protein ascitic fluids in cirrhotic patients are deficient in opsonic activity and are therefore predisposed to ascitic fluid infections [11]. It is believed that normal to high protein levels in the ascitic fluid are protective against SBP unless patients are exposed to a particularly virulent organism such as *Salmonella spp*. [11]. In our patient, total protein in the ascitic fluid was 1.6 g/dL, which confirmed previous reports that a low protein level may contribute to developing SBP secondary to *Salmonella spp.* [2]. However, other reports in both cirrhotic and non-cirrhotic patients have shown SBP secondary to *Salmonella spp.* to occur even with relatively normal to high ascitic fluid protein levels [8,12,13], specifically when patients are immunocompromised and unable to mount a proper immune response. One case report by Woolf et al. described two non-cirrhotic patients who developed SBP secondary to *Salmonella spp.*, despite ascitic protein levels greater than 2.5 g/dL [13]. In other published case reports, an immunocompromised state was believed to be a predisposing factor for SBP secondary to *Salmonella spp.*, and this included diabetes mellitus, malignancies, and human immunodeficiency virus (HIV) [2,3,5]. Our patient’s diagnosis with metastatic HCC along with the recent treatment with chemotherapy renders him immunocompromised and thus susceptive to *Salmonella spp.* induced SBP.

Some studies have reported that SBP secondary to *Salmonella spp.* could be asymptomatic, with a low PMN cell count in the ascitic fluid [14], which could contribute to a delay in the diagnosis in those patients until confirmation via an ascitic fluid culture [3,4,6]. Garcia et al. reported on three patients that were found to have *Salmonella enteritidis *SBP, two of whom died, one before treatment was started and the other patient on his second week of hospitalization [7], shedding light on the high mortality and poor prognosis associated with this disease entity. Therefore, if an infection is suspected, it is vital to attain ascitic cultures and start prophylactic treatment for SBP promptly despite low PMN cell counts in the ascitic fluid, until culture results are finalized. In our patient, SBP secondary to *Salmonella spp.* was diagnosed via both a high PMN count and positive cultures, with infection resolution and treatment efficacy evident on repeat ascitic fluid analysis.

## Conclusions

This is a rare occurrence of *Salmonella enteritica* isolated from ascitic fluid in a patient with decompensated cirrhosis. The patient’s history of metastatic HCC may reflect a potentially immunocompromised state, a common risk factor among similar reported cases. This case highlights the importance of recognizing *Salmonella spp.* as a possible pathogen in patients with SBP, and the importance of maintaining a wide differential of possible, less commonly seen pathogens in similar immunocompromised patients. Routine ascitic fluid culture should be performed in patients suspected of having SBP, with prompt initiation of IV antibiotics and de-escalation of treatment once ascitic cultures are finalized. These patients will require daily oral antibiotics for SBP prophylaxis.
